# Does the digital finance revolution validate the Environmental Kuznets Curve? Empirical findings from China

**DOI:** 10.1371/journal.pone.0257498

**Published:** 2022-01-13

**Authors:** Kaiyang Zhong

**Affiliations:** School of Economic Information Engineering, Southwestern University of Finance and Economics, Chengdu, China; China University of Mining and Technology, CHINA

## Abstract

In recent years, digital finance has become a crucial part of the financial system and reshaped the mode of green finance in China. Digital finance has brought certain impact on economic growth, industrial structure, and resident income, which may affect pollution. The nexus of digital finance and environment in China is thus worth exploring. By revising the traditional Environmental Kuznets Curve model with income inequality variable, this paper decomposes the environmental effects of economic activities into income growth effect, industrial structure effect and income inequality effect, and use panel data of China’s provinces to conduct an empirical analysis. The results reveal the following: (1) the Environmental Kuznets Curve is still valid in sample, and digital finance can reduce air and water pollution (as measured through SO_2_ and COD emission) directly; (2) in the influence mechanism, digital finance can alleviate income inequality and promote green industrial structure, thus reducing pollution indirectly, but the scale effect of income growth outweighs the technological effect, which increases pollution indirectly; and (3) digital finance has a threshold effect on improving the environment, then an acceleration effect appears after a certain threshold value. From the regional perspective, digital finance development in eastern regions is generally ahead of central and western regions, and the effects of environmental improvement in the eastern regions are greater. According to the study, this paper suggest that digital finance can be an effective way to promote social sustainability by alleviating income inequality and environmental sustainability by reducing pollution.

## 1. Introduction

In recent years, digital finance has become a crucial part of the financial system. Digital finance refers to the digitalization of the financial industry in general [1]. The McKinsey Report regarded digital finance as “financial services delivered via mobile phones, the internet or cards” [2]. Similarly, some scholars proposed that digital finance can be described as financial services delivered through mobile phones, personal computers, the Internet, or cards linked to a reliable digital payment system [3]. According to a report released by the Digital Finance Institute of Peking University in China, digital finance is a mode in which financial institutions and Internet companies apply digital technology to offer financing, payment, investment, and other new financial services [4]. Although until now there has been no universally accepted definition of digital finance, generally speaking, it is more like comprehensive practical activities integrating financial services and Internet technologies.

Since the Ant Financial Services Group (affiliated financial enterprise of Alibaba Group) launched Yuebao (a kind of online financial management) in 2013, China’s digital finance has developed vigorously, and the scales of third-party payment, online loans, digital insurance, and digital currency have ranked at the top globally [4]. According to the 43th China Statistical Report on Internet Development published by the China Internet Network Information Center (CINIC), at the end of 2018, the mobile network payment users had reached 583 million, and the Internet wealth management users had reached 150 million. The I-Research Group reported that the scale of China’s third-party mobile payment transaction had amounted to 190.5 trillion yuan (about USD 31 trillion) in 2018. Digital finance, obviously representing the future direction of the financial industry development, has covered business of credit, loan, and foreign exchange in China [4], and transformed China’s economy in an unprecedented way.

Although the relationship between economic activities and environmental pollution has been studied extensively [5, 6], the research regarding the impact of digital finance on the environment is still blank. There are two important aspects that should be discussed. One important aspect is that digital finance can influence inclusive growth. Digital finance has become a vital means to alleviate socio-economic inequality and push a more prosperous economy, thus leading to inclusive growth [7, 8]. Since inclusive growth can impact environment via increased income growth [9–10] and reduced inequality [11–16], such impact mechanisms of digital finance on the environment should be worth exploring. In empirical analyses, the Environmental Kuznets Curve (EKC) is regarded as a classic model to analyze the nexus between income growth and pollution [17], and there has been some revised EKC models that explored the impact of income inequality on pollution [18–22]. Therefore, EKC can be used as the basis of analysis method.

Another important aspect is that digital finance can influence the environment through the Internet. Internet usage can arouse the public’s attention to the environment [23–27], and digital finance can play a role in this. Take Ant Financial Services Group in China for example. This group has launched a personal carbon account called Ant Forest on its Alipay platform. Low-carbon buying behaviors can be recorded in the form of point calculations and converted into the cultivation of virtual trees (real trees will be planted in the desert equally). This digitally driven green innovation had received much attention and recognition from the United Nations Environment Programme (UNEP). In April 2019, Alipay officially announced that Ant Forest users had reached 500 million, and 100 million real trees had been planted. A similar case is happening with the German search engine Ecosia, because every user’s browsing is equivalent to planting a tree, and the company donates 80% of its search advertising profits to tree planting. These new modes combined with the Internet have brought great changes to green concepts.

Therefore, does the digital finance revolution validate the EKC? What is its impact mechanism? Clarifying these issues will help us uncover the digital finance’s impact on the environment. The main aim is to set up a revised EKC model to study the nexus between digital finance and environment, filling the gaps in current research. Based on existing studies [28–30], this paper will introduce an income inequality variable into the EKC model. This paper will also have some new designs according to the research purpose: (1) The variable of digital finance is also incorporated in order to study its direct environmental effects; (2) It not only tests the direct effect of digital finance on the environment, but also tests the indirect effect of digital finance on the environment by decomposing the environmental effects. Besides, this paper will apply adequate robustness tests and make more detailed analyses on the variables that measure EKC. Finally, this paper will offer suggestions for collaborative promotion of digital finance and environmental governance.

## 2. Literature review

At present, there is a lack of studies on digital finance and environmental pollution. This paper intends to take into account the digital nature and inclusiveness of digital finance, as well as their links with economic activities, to make a relevant literature summary.

### 2.1. Digital finance, income growth, and income inequality

Fisrt, this paper discusses digital finance and income growth. The development of the Internet had a huge impact on the transformation of business models, not only changing the face-to-face transaction process in the original traditional business model, but also reducing the transaction costs of financial services and significantly improving financial efficiency [31], thus solving the diseconomies of scale in traditional finance and promoting income growth [32]. Ozili (2018) elaborated on this from the perspective of economic and consumer activities, proposing that digital finance could boost the gross domestic product (GDP) of digital economies by offering a diverse range of financial products and services (especially credit facilities), and spur aggregate expenditure, thereby improving income [3]. Beck et al. (2018) used quantitative dynamic general models to conduct empirical research on mobile payment applications in Kenya’s digital finance, giving further proof that digital finance promoted income growth [9]. Li et al. (2020) used panel data from the China Household Finance Survey (CHFS) and an ordinary least squares (OLS) model to prove that inclusive digital finance could promote income growth by improving the penetrability of financial services and relieving residents from liquidity constraints [7]. Using a sample of 72 countries and applying dynamic generalized method of moments (GMM) estimation, Cheng et al. (2020) found that the interaction effects between information and communication technology (ICT), and financial development on income growth were positive [10].

Second, this paper discusses digital finance and income inequality. Another vital advantage demonstrated by digital finance was supporting the development of inclusive finance, which impacted income inequality for individuals [26]. In the past, Shaw (1973) noted the impact of finance on the income inequality and believed that financial development was an important means of narrowing the urban–rural income inequality [33]. A number of studies (e.g., [34–36]) supported the same results. With the development of information technology and Internet technology in recent years, some scholars have conducted research on the impact of financial development on income inequality in the digital context. Tchamyou et al. (2019) applied tests to 48 African countries and found that ICT reduced income inequality through formal financial sector development and formalization [15]. Mushtaq and Bruneau (2019) used a sample of 62 countries and found that the association of ICT diffusion with financial inclusion could help alleviate poverty and inequality; furthermore, ICT used as an instrument for financial inclusion accelerated such effects, therefore it could stimulate financial inclusion by advancing digital finance [16]. Zhong and Jiang (2021) offered empirical evidence in China, showing that Internet finance tended to weaken the exclusiveness of traditional finance, by reducing the asymmetry in traditional financial markets [37]. However, some scholars put forward different points of view. For example, Kar (2011) used data from 426 institutions in 81 countries for research and found that the relationship between inclusive finance and narrowing the urban–rural income inequality was not obvious [38].

### 2.2. Income growth, income inequality, and environment

There are extensive studies on the relationship between income growth and the environment using the method of EKC. Grossman and Krueger (1991) provided such a classic analytical model [17]. Based on Kuznets’ research theory on income distribution and income growth [39], they quantitatively analyzed the relationship between per capita income and environmental pollution indicators, and found that income growth and pollution emissions showed an inverted U curve relationship. Panayotou(1993) developed this finding into the EKC [40]. In follow-up studies, the EKC was used to describe the relationship between the economy and the environment, but those studies presented different opinions: some supported the EKC [41–43], while others had the opposite result [44–46]. Why did this happen? Harbaugh et al. (2002) [47] and Friedl et al. (2003) [48] questioned the robustness of the EKC; Cole et al. (2003) found that different pollutants also made an important difference [49]; Halkos (2012) argued that EKC might not be optimal if environmental critical loads were crossed irreversibly [50]; and Chen et al. (2019) suggested that it was necessary to distinguish less horrible pollution situations from more horrible ones, which required different strategies [51].

Some scholars sought to explore the relationship between income inequality and the environment by the extended EKC. Related research could be roughly divided into three categories. First, some literatures emphasized applications of econometric methods. Using a sample of Turkey and the ARDL test, Uzar and Eyuboglu (2019) showed that the EKC was valid in Turkey, and the Gini coefficient was the Granger-cause of CO_2_ emissions [22]. A similar method and result can be seen in [28]. However, these tests were described more statistically, rather than based on theoretical research. Second, some literatures put emphasis on simply adjusting the EKC model variables. For example, Chen et al. (2020) used the Gini coefficient as proxy variable for income inequality and combined it with per capita income to conduct tests, finding that the EKC hypothesis was valid, and for G20 countries, income inequality in most developed countries hardly affected CO_2_ emissions, while more equal income distribution in developing countries favored a reduction in CO_2_ emissions [29]. Third, some literatures focused on the research objects. For instance, Dong and Hao (2018) used the urban–rural income gap as income inequality and per capita electricity consumption as the explained variable to study whether income inequality affected electricity consumption in China [30].

### 2.3. Internet and environment

Sections 2.1 and 2.2 refer to the inclusive characteristics of digital finance, but in addition, it also has Internet and digital features that impact the environment, so this paper sort out the literature on the latter. Lim and Lee(2012) proposed that utilising Internet technologies to offer access to environmental information could put community pressure on polluters [52]. Salahuddin et al. (2016) tested the relationship between Internet usage and CO_2_ emissions in OECD countries, and reported that although there was a positive significant relationship in the long run [23]. In another sample of 21 sub-Saharan African countries, the empirical results indicated that mobile phone and Internet penetration had direct positive effects on CO_2_ emissions [53]. Zhang and Meng (2019) tested the EKC with Internet penetration in 115 countries, indicating that the EKC existed and Internet penetration pushed a decrease in CO_2_ emissions [27]. Zhang et al. (2019) conducted research on the Internet and residents’ environmental awareness in China, and reported that increased online content frequency reduced residents’ satisfaction with the government’s environmental protection [54]. Gong et al. (2020) investigated the effect of Internet use on pro-environmental behavior in China and reported a significant positive relationship, suggesting that pro-environmental behavior can be encouraged by improving Internet access [55]. Hsu et al.(2020) utilized the app’s citizen reports in Guangzhou(a big city in China) to find that the reports were helpful to significantly improving water quality [56]. However, some scholars had argued that Internet penetration did not significantly influence individual sustainable consumption behaviors, but substantially enhanced the transition from pro-environmental attitudes to sustainable behaviors [26].

This paper will study the environmental effects of digital finance based on the existing literature, and the steps of analysis are as follows: first, it revises the EKC model in the context of digital financial development and explores the direct effects of all variables; second, it tests the influence mechanism of digital finance on the environment, to explore the indirect effects; and third, it checks whether there are heterogeneous effects. In view of the shortcomings of current literature, it will improve the following: first, to avoid simply using econometric methods to analyze the effects of digital finance on pollution, it intends to set up the theoretical basis of the revised EKC; second, it uses at least two pollutants, including air and water pollutants, and conducts adequate robustness tests; and lastly, it makes more detailed analyses on the variables that measure EKC.

## 3. Research design

### 3.1. Theoretical basis

As introduced in the above literature review, although some scholars had applied the adjusted EKC to study the impact of income inequality on pollution, they simply added the variable of income inequality to the EKC model, so the shortcomings were obvious: first, those studies did not analyze specific causes beyond income, but simply described whether the EKC was established, but the impact of income growth on pollution can be attributed to the effects of scale expansion and technological progress, which should be further analyzed; and second, those studies usually stopped at the repeated testing of a model, but there was no further mechanism testing, which cannot be applied to the research objects. Therefore, this paper intends to integrate the research by Grossman and Krueger (1991) [17], Antweiler et al. (2001) [57], and Cole and Elliott (2003) [49] to establish the theoretical basis of research.

The great contribution of Grossman and Krueger (1991) was to propose a theoretical basis to analyze the environmental effects of economic growth [17]. They decomposed the environmental effects of economic growth into scale, technological, and structural effects. With the scale effect, when the nature of the economy remains unchanged, the expansion of output scale will accelerate resource consumption and bring more pollution emissions. With the income effect, as income increases, people will require higher standards of environmental regulation, prompting progress in environmental protection technology. The structural effect refers to the expansion or contraction of pollution-intensive industries in a region based on relative comparative advantage, and changes of industrial structure will affect the pollution level. This paper follows this theoretical analysis with some alteration.

Antweiler et al. (2001) further provided a micro-theoretical model for the analysis of Grossman and Krueger [57]. The relationship between the three effects is as follows:

$$\hat{z}=\pi_{1}\hat{S}-\pi_{2}^{}\hat{R}+\pi_{3}\hat{\kappa }$$ (1)

where ^ represents the differential form ($\hat{X}=dX/X$); *π*_*i*_ is the coefficient value and *π*_*i*_ > 0; $\hat{S}$ is the scale effect, which has positive impact on pollution; $\hat{R}$ is the technical effect, which has a negative impact on pollution; and $\hat{\kappa }$ is the structural effect, representing pollution-intensive industries, which has a positive impact on pollution. In empirical research [49], the scale and technical effects are combined, replaced by GDP per capita. The first and square terms of GDP per capita are the empirical basis of the EKC. At the catch-up phase of economic growth, the scale effect is generally greater than the technological effect, and when economic growth reaches a new stage, the scale effect may be smaller than the technical effect.

Model (1) solves the problem of the relationship between total income growth and pollution, but does not address the relationship between income structure and pollution. It can be seen in previous literature reviews that income inequality may have an impact on environmental pollution. When analyzing a country’s economic growth and pollution, income distribution should be an important aspect [5, 6], especially for China, which has developed into a vital economy in the world but is still a developing country at the per capita income level, so the impact of the income gap on environmental pollution should not be neglected. Therefore, this paper incorporates the income inequality variable into the EKC model. Adding the income inequality variable (I) and digital finance variable (f) to Eq (1), I can get

$$\hat{z}=\pi_{1}\hat{S}-\pi_{2}^{}\hat{R}+\pi_{3}\hat{\kappa }\pm \pi_{4}\hat{I}\pm \pi_{5}\hat{f}$$ (2)

where $\pi_{1}\hat{S}-\pi_{2}^{}\hat{R}$ represents the environmental effects brought by income growth, called the income growth effect; $\pi_{3}\hat{\kappa }$ represents the comparison between pollution-intensive and clean industries, called the industrial structure effect; $\pi_{4}\hat{I}$ represents the effects of income inequality differentiation, called the income inequality effect; and $\pi_{5}\hat{f}$ represents the direct effect of digital finance.

With further derivation of Eq (2) by the digital finance variable (*f*), I can get

$$\frac{dz}{df}\frac{f}{z}=\pi_{1}\frac{dS}{df}\frac{f}{S}-\pi_{2}\frac{dR}{df}\frac{f}{R}+\pi_{3}\frac{d\kappa }{df}\frac{f}{\kappa }\pm \pi_{4}\frac{dI}{df}\frac{f}{I}\pm \pi_{5}$$ (3)

Eq (3) reveals the indirect environmental effects of digital finance:

1. $\pi_{1}\frac{dS}{df}\frac{f}{S}-\pi_{2}\frac{dR}{df}\frac{f}{R}$ represents the indirect effect of digital finance on the environment by affecting income growth;
2. $\pi_{3}\frac{d\kappa }{df}\frac{f}{\kappa }$ represents the indirect effect of digital finance on the environment by affecting the industrial structure; and
3. $\pi_{4}\frac{dI}{df}\frac{f}{I}$ represents the indirect effect of digital finance on the environment by affecting income inequality.

### 3.2. Model setting

The benchmark panel model is established as follows:

$$lnY_{it}=\alpha_{0}+\alpha_{1}Index_{it}+\alpha_{2}PGDP_{it}+\alpha_{3}PGDP_{it}^{2}+\alpha_{4}Theil_{it}+\alpha_{5}Industry_{it}+\varepsilon_{it}$$ (4)

where:

1. The dependent variable *Y*_*it*_ represents pollution indicators. This paper takes sulfur dioxide (SO_2_) emission and chemical oxygen demand (COD) emission as pollution indicators, which are two of the main pollutants in China. Referring to the research of Antweiler et al. [57], this paper adopts the natural logarithm form for pollution indicators, which further reduce regional differences and facilitates comparison of different pollution indicators.
2. The explanatory variable *Index*_*it*_ represents the digital financial level. This paper uses data of the Peking University Digital Inclusive Financial Index (2011–2015) compiled by the university’s Digital Finance Institute, currently China’s most authoritative digital financial index. This paper introduces it more in Section 3.3.1. In order to avoid the value of the coefficient being too small, the digital financial data here is divided by 100.
3. The explanatory variable *PGDP*_*it*_ represents the income growth effect, and its value is GDP per capita. For *PGDP*_*it*_, when the coefficient of the first term is positive and the coefficient of the second term is negative, the relationship between economic growth and environmental pollution is an inverted U and the traditional EKC is proved [49].
4. The explanatory variable *Theil*_*it*_ represents the income inequality effect. The Theil index considers the factors of population changes and decomposes the urban–rural income gap into intra-group and inter-group gaps, so that their changes can be better measured [58–60]. The calculation formula is
$$Theil_{it}=\sum_{j=1}^{j=2}\left(\frac{y_{ijt}}{y_{it}}\right)ln(\frac{y_{ijt}}{y_{it}}/\frac{x_{ijt}}{x_{it}})$$ (5)
  where j = 1 represents urban area and j = 2 represents rural area. *Theil*_*it*_ represents the Theil index of region i at period t; *y*_*i1t*_ represents the total urban income of region i at period t (per capita disposable income of urban residents multiplied by total urban population); *y*_*i2t*_ represents the total rural income of region i at period t (per capita disposable income of rural residents multiplied by total rural population); *y*_*it*_ represents the sum of urban and rural incomes of region i at period t; *x*_*ijt*_ represents the population of region i in urban area (j = 1) or rural area (j = 2) at period t; and *x*_*it*_ represents the total population of region i at period t.
5. The explanatory variable *Industry*_*it*_ represents the industrial structure effect. Some scholars argued that, under the framework of EKC, pollution emissions kept low in the agrarian economies, increase in the process of industrialization, and decrease again in a service-based economy [61, 62]. In China, many pollution-intensive industries belong to the secondary industry, and the tertiary industry is relatively less polluted [63]. So, this paper measures industrial structure effect by the ratio of the added value of the secondary industry to that of the tertiary industry.

### 3.3. Data

According to data availability, with the period of the Peking University Digital Inclusive Financial Index (2011–2015), this paper used provincial panel data from 2011 to 2015 and a cross-section of 31 provinces in China (due to the data source, Hong Kong, Macau, and Taiwan were not included in sample). Unless otherwise specified, the data came from the China National Statistical Yearbook. In order to alleviate the influence of inflation, this paper adjusted per capita GDP (PGDP) by the Consumer Price Index (CPI), with the base period fixed as 2011. The variables Theil and Industry were the calculation results; the numerator and denominator simultaneously eliminated the inflation factor in the calculation process, so this paper did not deal with inflation factor further.

#### 3.3.1. Digital finance index

The digital finance index data came from the report released by the Digital Finance Institute of Peking University. To scientifically and accurately portrayed the development status of China’s inclusive digital finance, the Digital Finance Institute, the Shanghai New Financial Research Institute, and the Ant Financial Group formed a joint research group using massive data on inclusive digital finance to compile the index. The data were from Ant Financial Group and other representative Internet financial companies or third-party organizations. According to the attributes of Internet financial business, the report divided businesses into five major Internet sections: payment, credit, insurance, investment management, and credit reporting. For the indicator system, the report set up the index with three dimensions of coverage breadth, depth of use, and digital support services, including a total of 24 indicators. The report treated the indicators with different properties and measurement units in a dimensionless manner; that was, they transformed the values of different measurement units into the same measurement value, assigned a weight for each index and calculated them to obtain the final index value. Therefore, the index is a relative value for comparison. The index data are shown in Table A1 of S1 Appendix.

#### 3.3.2. Descriptions of variables and data

This paper provides statistical description of the data for each variable, which is shown in Table 1. According to the data description, the differences of pollution emissions between provinces are great. After the pollution emissions are processed in logarithmic form, the differences have been greatly reduced.

**Table 1:** Descriptions of variables and data.

Variable	Meaning	Obs	Mean	Std. Dev.	Min	Max
SO_2_	SO_2_ emissions (10,000 tons)	155	65.89	41.12	0.42	182.74
LnSO_2_	Natural logarithm of emissions	155	3.81	1.18	–0.86	5.20
COD	COD emissions (10,000 tons)	155	76.09	49.44	2.58	198.25
LnCOD	Natural logarithm of emission	155	4.02	0.92	0.94	5.28
Index	Digital finance index reported by Digital Finance Institute of Peking University	155	138.95	67.08	16.22	278.11
PGDP	Per capita GDP (10,000 yuan)	155	4.44	1.99	1.64	9.84
Theil	Theil index measuring income inequality between urban and rural areas	155	0.10	0.04	0.02	0.22
Industry	Ratio of added value of secondary industry to that of tertiary industry	155	1.15	0.34	0.24	1.92

Note: Index data are from Peking University Digital Inclusive Financial Index (2011–2015) compiled by the university’s Digital Finance Institute, and data of other variables (including original variables for calculation) are from the China National Statistical Yearbook.

## 4. Model regression results and analysis

### 4.1. Benchmark model regression results and analysis

This paper conducted regression on the benchmark model. For model selection, first, this paper used tests to choose mixed regression (pooled OLS) model or individual fixed effect (FE) model. From the results of the FE, the F-statistic was significant at the level of 1%, and the pooled OLS model was rejected. Then this paper used tests to choose the FE or random effect (RE) model; the Hausman test statistic was significant at the level of 1%, so the RE model was rejected. This paper conducted further tests to choose the FE or individual time fixed-effect model by testing the joint significance of all dummy annual variables, as the F-statistic was not significant within 10% (for SO_2_, F-statistic = 1.86, p = 0.12; for COD, F-statistic = 1.21, p = 0.31), thus the original hypothesis of "no time effect" could not be rejected and the individual FE model was chosen. Moreover, considering that the sample data had a short time frame, it was more suitable to use individual FE model in this study. The regression results are shown in Table 2.

**Table 2:** Regression results of benchmark model.

Variable	lnSO_2_	lnCOD
	FE	FE
Index	−0.0382* (1.95)	−0.0351*** (3.57)
PGDP	0.1509*** (3.03)	0.1014*** (4.06)
PGDP^2^	‒0.0160*** (5.63)	‒0.0102*** (7.16)
Theil	1.6205*** (3.68)	0.6104*** (2.76)
Industry	0.0758** (2.06)	0.0673*** (3.64)
Constant	3.3219*** (19.32)	3.7234*** (43.14)
R-squared	0.7237	0.8292
F-test	2744.57***	6575.22***
Hausman test	16.91***	19.57***
Obs	155	155

Note: t-values are reported in parentheses.

* p < 0.1;

** p < 0.05;

*** p < 0.01.

According to Table 2, for regression of the two pollution indicators, the coefficients are both significant, and their values are close and signs are consistent, which shows good robustness of these models. Next, this paper analyzes the regression results of each variable:

1. The Index coefficient is significantly negative, indicating that development of digital finance can promote reduced pollution emissions, and the direct effect of digital finance on the environment is positive. First, the development of digital finance is closely related to the development and popularization of the Internet [25–27], and the Internet can improve environmental quality to a certain extent as illustrated in the literature review. In 2015, the number of Internet users in China was 688 million, and the Internet penetration rate reached 50%. Second, the development of digital finance contributes to environmental education. The Ant Financial Services Group, which launched Ant Forest, a personal carbon account that encouraged people to practice low-carbon living, is a good example. Third, digital finance is a platform that can encourage innovations, which can contribute to dematerialization (digital currency, digital financial contracts, etc.) and reduced greenhouse gas emissions (online financial transactions, mentoring, training, etc.).
2. For PGDP, the coefficient of its first term is positive and the coefficient of its second term is negative, indicating that in the stage of the period 2011–2015, income growth and pollution emissions showed an inverted U relationship. In previous studies on the selection of multiple pollutants, the EKC was difficult to validate [64–66], but it is valid in this study. The main reason may be as follows: on one hand, this paper adopted the natural logarithm form for pollution indicators, which further reduced regional differences and data sensitivity; on the other hand, the range of 2011 to 2015 was the implementation period of the National Environmental Protection (Twelfth Five-Year Plan) in China, so SO_2_ and COD emissions might have the same change trend under the regulation of emission targets. In the results, the inflection points of SO_2_ and COD emissions are approximately 75,000 yuan (about USD 12,000) and 50,000 yuan (about USD 8000), respectively. Four and ten provinces surpass the inflection point respectively. Most provinces cannot surpass the inflection point and are distributed on the left side of it.
3. The Theil coefficient is significantly positive, so widening the income gap increases pollution. There may be several reasons behind this. First, as the urban-rural income gap widens, some labor-intensive industries will be transferred from urban areas to rural areas, and such industries are more polluted. Second, as the income gap widens, people at lower income levels (especially in rural areas) will have lower requirements for environmental protection in order to survive and develop. The rural population is still relatively large in China, and in 2015, this number was 600 million, accounting for about 50% of the total population. The environmental pressure brought by income inequality is still great. These two factors may play a role at the same time. Therefore, even if pollution-intensive industries are not reduced in the rural area, the increase in the relative income of rural residents may also contribute to the environmental improvement. It can also be understood from the perspective of EKC: the widening of the income gap often requires vigorous economic growth to solve it, and the scale effect on the environment is greater than the technological effect, thus increasing pollution.
4. The Industry coefficient is significantly positive, indicating that as the proportion of the secondary industry increases, environmental pollution also increases. According to the China National Statistical Yearbook, in 2015, the top five industries that emitted the most SO_2_ were gas production, smelting and pressing of ferrous metals, smelting and pressing of non-ferrous metals, manufacture of medicines, and manufacture of metal products, which were capital-intensive secondary industries. However, China’s tertiary industry has developed rapidly and its proportion has been continuously increasing; the proportion of the tertiary industry exceeded 50% for the first time in 2015. Most of the growth momentum of the tertiary industry comes from small and medium enterprises (SMEs), which account for 90% of national enterprises and more than 80% of employment. Especially with the development of China’s e-commerce industry, many SMEs complete transactions through the Internet, which helps to improve the environment.

### 4.2. Robustness test

#### 4.2.1. Changing the form of pollution indicators

The forms of pollution indicators mainly include total (total emission), per capita (total emission divided by total population), and intensive (total emission divided by GDP) [49]. Previous literature generally used one or two forms. In fact, different pollution indicators may have different performance. In order to further test the robustness, this paper replaced total form with per capita and intensive form to conduct regression. The results are shown in Table 3.

**Table 3:** Regression results of per capita form and intensive form.

Variables	lnSO_2_	lnCOD	lnSO_2_	lnCOD
	per capita form	per capita form	intensive form	intensive form
	FE	FE	FE	FE
Index	‒0.0575*** (3.14)	‒0.0543*** (6.67)	‒0.1102*** (5.29)	‒0.1071*** (9.85)
PGDP	0.1845*** (3.96)	0.1350*** (6.51)	‒0.2445*** (4.61)	‒0.2940*** (10.63)
PGDP^2^	‒0.0189*** (7.10)	‒0.0131*** (11.07)	0.0026 (0.89)	0.0084*** (5.36)
Theil	1.7261*** (4.18)	0.7160*** (3.90)	2.0283*** (4.32)	1.0183*** (4.16)
Industry	0.0619* (1.80)	0.0534*** (3.48)	0.0150 (0.38)	0.0064 (0.32)
Constant	‒4.8400*** (30.08)	‒4.4385*** (62.00)	‒5.9000*** (32.24)	‒5.4985*** (57.58)
R-squared	0.8074	0.9192	0.9519	0.9832
F-test	1299.75***	1712.08***	2467.80***	5538.44***
Hausman-test	14.44**	26.66***	17.34***	20.04***
Obs	155	155	155	155

Note: t-values are reported in parentheses.

* p < 0.1;

** p < 0.05;

*** p < 0.01.

It can be seen from Table 3 that the signs of all variables remain unchanged, the coefficient values are significantly improved, and the significance remains good. For the regression results of the intensive form, PGDP, excluding the scale effect, represents the technical effect of economic growth. This reveals that people will require higher standards of environmental regulation, which basically confirms Grossman and Krueger’s assumption about technological effects [17]. As the scale effect of increasing pollution exceeds the technical effect of reducing pollution, ultimately leading to economic growth with increasing pollution, the PGDP coefficient is positive for the total and per capita forms. The results show that China is still at the stage of chasing economic growth.

#### 4.2.2. Alleviating the endogeneity problem

In this section, this paper uses further tests to reduce the endogeneity problem. Considering the short sample time, this paper lagged all explanatory variables by one period and used the FE model for regression. The results show that the signs of the variables under the two pollution indicators remain unchanged, the significance is better, and the coefficient values of most variables are improved. Regression results can be seen in the Table A2 of S1 Appendix.

#### 4.2.3. Adjusting the sample range

The time period 2011 to 2015 was the implementation period of the National Environmental Protection (Twelfth Five-Year Plan) in China. In order to achieve the goals of pollution reduction, government departments were likely to abnormally reduce pollution emissions in 2015, thereby creating a braking effect. The braking effect might make the results of EKC unreliable. So, this paper deleted the data of 2015 from the sample and conducted empirical regression based on data from 2011 to 2014. The results show that the regression results are basically consistent with the benchmark model. The EKC is still valid. Therefore, the braking effect can be excluded in the sample. Compared with the model of 2011–2014, in the model of 2011–2015, the absolute values of the digital finance coefficients are smaller for both pollutants (for SO_2_, from 0.0575 to 0.0382; for COD, from 0.0376 to 0.0351), but they become closer between the two pollutants. Regression results can be seen in the Table A3 of S1 Appendix.

### 4.3. Intermediary effect test

In order to verify the mechanism of digital finance affecting environmental pollution, this paper builts the following regression model to test the intermediary effect:

$$lnY_{it}=\mu_{0}+\mu_{1}Index_{it}+\sum \mu_{k}X_{kit}+\varepsilon_{it}$$ (6)

$$Mediator_{it}=\varphi_{0}+\varphi_{1}Index_{it}+\sum \varphi_{k}X_{kit}+\varepsilon_{it}$$ (7)

$$lnY_{it}=\eta_{0}+\eta_{1}Index_{it}+\eta_{2}Mediator+\sum \eta_{k}X_{kit}+\varepsilon_{it}$$ (8)

Where *Mediator*_*it*_ is an intermediary variable, including income growth effect (PGDP), income inequality effect (Theil), and industrial structure effect (Industry); and *X*_kit_ is a controlled variable. The test procedure is as follows: First, coefficient *μ*_1_ in model (6) should be statistically significant. Second, in the case where *ϕ*_1_ and *η*_2_ are both significant, if *η*_1_ is statistically significant and the value is smaller than *μ*_1_, it means that there is a partial intermediary effect, and if *η*_1_ is not statistically significant, it means that there is a complete intermediary effect. Third, if either *ϕ*_1_ or *η*_2_ is not statistically significant, this paper need to further determine whether there is an intermediary effect by the Sobel test. In models (6)–(8) for testing intermediary effects, model (8) is actually the benchmark model (4). Table 4 lists the regression results of main variables in models (6) and (7), and regression results of model (8) can be seen in Table 2.

**Table 4:** Intermediate effect regression results.

	Income growth effect			Income inequality effect			Industrial structure effect		
	ln *Y*_i*t*_ = SO_2_	ln *Y*_i*t*_ = COD	PGDP	ln *Y*_i*t*_ = SO_2_	ln *Y*_i*t*_ = COD	Theil	ln *Y*_i*t*_ = SO_2_	ln *Y*_i*t*_ = COD	Industry
	model (6)	model (6)	model (7)	model (6)	model (6)	model (7)	model (6)	model (6)	model (7)
	FE	FE	FE	FE	FE	FE	FE	FE	FE
Index	−0.0762*** (6.23)	−0.0560*** (8.51)	0.6522*** (16.85)	−0.0572*** (2.89)	−0.0423*** (4.34)	−0.0117*** (3.00)	−0.0632*** (4.05)	−0.0573*** (7.06)	−0.3286*** (8.62)
other variables	−	−	−	−	−	−	−	−	−
R-squared	0.6171	0.7279	0.8632	0.6924	0.8183	0.7255	0.7139	0.8102	0.6424
F-test	2159.50***	4757.50***	299.24***	2569.24***	6487.36 ***	42.5***	3557.68***	6878.72***	34.12 ***
Hausman- test	19.79***	13.86***	77.87***	15.57***	19.00***	19.65***	8.11*	12.34**	34.98*

Note: t-values are reported in parentheses.

* p < 0.1;

** p < 0.05;

*** p < 0.01.

From the regression results of intermediary effects, the test results under the two pollution indicators are basically consistent. The coefficient *μ*_1_ of *I*n*dex*_*it*_ in model (6) and the coefficients of *ϕ*_1_ for the intermediary variables of PGDP, Theil, and Industry in model (7) are all significant, indicating that there are intermediary effects. In addition, the absolute value of the *I*n*dex*_*it*_coefficient in Table 2 is less than *μ*_1_ in Table 4, so it can be judged that there is partial intermediary effects. Let us analyze the economic significance of the intermediary effects:

1. The first relationship is that digital finance promotes PGDP, while PGDP is proved to increase pollution. First, digital finance can increase household consumption, smoothing household income fluctuations through the financial savings function, thus promoting economic growth. Second, digital finance can make it easier for enterprises to obtain more credit and other online financial support, thereby increasing production capacity. Third, digital finance has driven the development of mobile payment and e-commerce, which also drives economic growth. Lastly, by diversifying the risk of the real economy, the economic environment is more stable and enterprises are more confident in economic development, so they dare to invest and innovate in technology.
2. The second relationship is that digital finance alleviates income inequality, while income inequality is proved to increase pollution. One of the biggest problems faced by traditional finance in the past was that many rural residents, low-income residents, and SMEs lacked collateral or credit records, making it difficult to obtain financing. With the development of digital finance, the Internet and big data technology can solve such problems. Especially with the rise of rural e-commerce and the popularization of mobile payments, many agricultural products can be sold online, increasing farmers’ income. According to the China E-Commerce Report 2019 released by the National Ministry of Commerce, rural online retail sales reached 1.7 trillion yuan (about USD 250 billion).
3. The third relationship is that digital finance reduces the ratio of the secondary industry to the tertiary industry, while such reduction is proved to decrease pollution. This paper suggest that it can be understood from at least two aspects: First, digital finance has alleviated the financing difficulties of SMEs through big data technology, promoting innovation and entrepreneurship. SMEs account for more than 90% of the total enterprises in China, and most of them belong to the tertiary industry. Second, digital finance has promoted the resonant development of the Internet and e-commerce; for example, according to the China E-commerce Report 2019, e-commerce transactions reached 34.81 trillion yuan (about USD 5 trillion), which can spur the development of the tertiary industry.

The effects of the indirect influence on pollution by digital finance are summarized in Table 5. On the whole, digital finance has indirect effects on pollution through the income growth, income inequality, and industrial structure effects. Digital finance indirectly reduces pollution by alleviating income inequality and promoting green industrial structure, but the scale effect through income growth exceeds the technological effect, thus indirectly increasing pollution. Moreover, the digital finance is helping China to achieve the UN Sustainable Development Goals (eighth goal on decent work and economic growth; tenth goal on reduced inequality). Additionally, inclusive growth (promoting income growth and alleviating income inequality simultaneously) leads to less pollution than the pure income growth.

**Table 5:** Indirect effects of digital finance on environmental pollution.

Intermediary variables	Effect of Index (digital finance) on intermediary variables	Effect of intermediary variables on environment	Overall effect of Index on environment
PGDP	+	+	+
Theil	‒	+	‒
Industry	‒	+	‒

### 4.4. Heterogeneity test

The results of benchmark regression and robustness tests show that, on the whole, China’s digital financial development can reduce pollution and improve environmental quality. However, the index of digital finance varies greatly among provinces. For example, in 2015, Shanghai’s digital financial index was the highest, at about 278, and Tibet’s digital financial index was the lowest, at about 186. Is there a process of self-accumulation in the development of digital finance, and when it reaches a certain level, will there be a qualitative change? If this is the case, the economically backward areas should pay more attention to the accumulation effect of developing digital finance and implement a corresponding development strategy in accordance with the different stages. The problems mentioned above can be tested by a threshold panel model.

Taking digital finance as the threshold variable, this paper established a threshold panel model:

$$\begin{array}{l} lnY_{it}=\beta_{0}+\beta_{1}Index_{it}I(Index_{it}>\gamma )+\beta_{2}Index_{it}I(Index_{it}\leq \gamma )+\\ \beta_{3}PGDP_{it}+\beta_{4}PGDP_{it}^{2}+\beta_{5}Theil_{it}+\beta_{6}Indusry_{it}+\varepsilon_{it} \end{array}$$ (9)

where I(.) is the indicative function and *γ* is the estimated threshold value of Index, which is determined endogenously by sample data. The threshold effect test is performed first, affirming whether there are threshold points in the model. Threshold effect test results are shown in Table 6. The single threshold panel model passes the test at a significance level of 10%, while the double threshold model does not pass the significance test. Therefore, both pollution indicators have a single threshold effect.

**Table 6:** Threshold effect test results.

Pollution indicator	Threshold test	F-test	P-value	Test results	Threshold point of Index
SO_2_	Single threshold	13.44*	0.0860	Single threshold	202
	Double threshold	11.91	0.1620		
COD	Single threshold	12.73*	0.0900	Single threshold	239
	Double threshold	13.24	0.2000		

Note:

* p < 0.1.

From the regression results in Table 7, the digital finance index still significantly improves environmental pollution, and the improvement is amplified after a certain point, showing an accelerated effect: For SO_2_, when the digital finance index is greater than 202, the absolute value of the coefficient increases from 0.0315 to 0.0489; for COD, when the index is greater than 239, the absolute value increases from 0.0375 to 0.0488. This may be due to the accumulation effect of digital finance, which is more effective in making the economy move toward greener development. Judging from the changes in provinces where the digital finance index is below the threshold (Table 8), the number of central and western regions is significantly higher compared to the eastern regions. For the central and western regions where digital financial development lags behind, when faced with a new round of competition in the coordinated development of green finance and economic growth, they need to catch up with the eastern regions.

**Table 7:** Regression results of threshold model.

Variables	lnSO_2_	lnCOD
Index>202	‒0.0489** (2.56)	
Index≤202	‒0.0315* (1.66)	
Index>239		‒0.0488*** (4.45)
Index≤239		‒0.0375*** (3.89)
PGDP	0.1398*** (2.91)	0.0985*** (4.03)
PGDP^2^	‒0.0142*** (5.08)	‒0.0090*** (6.09)
Theil	1.7295*** (4.07)	0.6175*** (2.86)
Industry	0.0404 (0.27)	0.0662*** (3.66)
Constant	3.3558*** (20.25)	3.7119*** (43.95)
R-squared	0.6736	0.7258
F-test	2946.90***	6854.16 ***
Obs	155	155

Note: t-values are reported in parentheses.

* p < 0.1;

** p < 0.05;

*** p < 0.01.

**Table 8:** Changes in provinces below threshold point.

SO_2_(Index ≤ 202)				COD(Index ≤ 239)			
Year	Province	Eastern regions	Central-western regions	Year	Province	Eastern regions	Central-western regions
2011	31	11	20	2011	31	11	20
2012	31	11	20	2012	31	11	20
2013	28	8	20	2013	31	11	20
2014	26	6	20	2014	29	9	20
2015	5	1	4	2015	25	5	20

## 5. Conclusion and suggestion

Income growth and income distribution are two important aspects that affect environmental sustainability. At the same time, income distribution issues will also affect the sustainability of society. The EKC is an important model for analyzing the relationship between income and pollution, but previous analysis only focused on the overall impact of income variables in the statistical sense and did not explore reasons behind it, and they lacked an analysis of the impact mechanism.

Due to the digitality and inclusiveness, digital finance has a unique impact on the development of green financial mode and green economy. Digital finance not only has a direct impact on pollution based on the characteristics of the Internet, but also has indirect effects based on other factors. Therefore, studying the environmental effects of digital finance and applying it to improve the environment have great value. The contributions are mainly as follows: (1) considering the impact of income distribution on the environment, this paper revised the traditional EKC model by adding an income inequality variable, thus decomposing the environmental effects of economic activities into income growth, industrial structure, and income inequality effects; (2) this paper further added digital finance variable to the revised model, reassessed the environmental effects of economic growth in the context of digital financial development, and tested the influence mechanism; and (3) this paper checked whether there were heterogeneous effects of digital finance at different levels on the environment, and gained further understanding of the environmental effects of digital finance at the micro level.

As the development scale of China’s digital finance is at the forefront globally, this paper took China as an example to research environment effects of digital finance and got the following results: (1) The Environmental Kuznets Curve is still valid in the sample, and digital finance can reduce air and water pollution (as measured through SO_2_ and COD emission) directly. (2) By analyzing intermediary effects, it can be seen that digital finance can indirectly reduce pollution by alleviating income inequality and promoting green industrial structure; however, the scale effect of digital finance through income growth exceeds the technological effect, indirectly increasing pollution. (3) As shown by an empirical test of the threshold effect with a panel model, the improvement effect of digital finance on pollution will magnify when the digital finance index passes a certain point, showing an accelerated effect. Eastern regions have advantages in the development of digital finance to improve the environment compared to central and western regions.

Based on the study, this paper puts forward the following suggestions:

1. Based on China’s experience, digital finance can be a useful means for developing countries to solve financial problems, promote inclusive growth, and achieve the UN Sustainable Development Goals. With the big data technology and the Internet (especially mobile Internet), finance is easier to reach for individuals and SMEs, which promotes inclusive financial services. In addition, digital finance can offer contactless services through the Internet, which is especially important in the fight against COVID-19. Although the integration of digital technology and finance has different performances in different countries, in general, such integration will be a trend.
2. Digital finance can be developed to promote environmental sustainability. First, the main service targets of digital finance are individuals and SMEs, which can effectively alleviate the problem of income inequality, thereby promoting the environmental improvement. Second, digital finance can be developed to drive the development of the country’s tertiary industry that is less polluted. Third, digital finance promotes the popularization of the mobile Internet and raises the public’s environmental awareness. Therefore, digital finance can be an effective means of promoting environmental improvement through the use of the Internet. For example, mobile apps that track environmental conditions, such as factory pollution discharges and individual carbon footprints, can be greatly encouraged. Information, technology and digitalization can be integrated more deeply in environmental governance.
3. The gap in the development of digital finance among regions should be paid attention to. Due to gaps in Internet penetration and economic factors, the phenomenon of digital divide may exist. Therefore, regional difference should be considered by policymakers so as to prevent the development of digital finance from leading to a new round of injustice.

## Supporting information

S1 Appendix (DOCX)
