# Supplementary material for: Does the digital finance revolution validate the Environmental Kuznets Curve? Empirical findings from China
Source: PLoS One. 2022 Jan 13;17(1):e0257498. doi: 10.1371/journal.pone.0257498 (PMC8758091; doi:10.1371/journal.pone.0257498)
Supplement: S1 Appendix — (DOCX) [file pone.0257498.s001.docx]

Appendix

**Table A1.** Digital finance index.

| **Province** | **Region** | **2011** | **2012** | **2013** | **2014** | **2015** |
| --- | --- | --- | --- | --- | --- | --- |
| Beijing | Eastern | 79.41 | 150.65 | 215.62 | 235.36 | 276.38 |
| Tianjin | Eastern | 60.58 | 122.96 | 175.26 | 200.16 | 237.53 |
| Hebei | Eastern | 32.42 | 89.32 | 144.98 | 160.76 | 199.53 |
| Shanxi | Central | 33.41 | 92.98 | 144.22 | 167.66 | 206.3 |
| Neimenggu | Central | 28.89 | 91.68 | 146.59 | 172.56 | 214.55 |
| Liaoning | Eastern | 43.29 | 103.53 | 160.07 | 187.61 | 226.4 |
| Jilin | Central | 24.51 | 87.23 | 138.36 | 165.62 | 208.2 |
| Helongjiang | Central | 33.58 | 87.91 | 141.4 | 167.8 | 209.93 |
| Shanghai | Eastern | 80.19 | 150.77 | 222.14 | 239.53 | 278.11 |
| Jiangsu | Eastern | 62.08 | 122.03 | 180.98 | 204.16 | 244.01 |
| Zhejiang | Eastern | 77.39 | 146.35 | 205.77 | 224.45 | 264.85 |
| Anhui | Central | 33.07 | 96.63 | 150.83 | 180.59 | 211.28 |
| Fujian | Eastern | 61.76 | 123.21 | 183.1 | 202.59 | 245.21 |
| Jiangxi | Central | 29.74 | 91.93 | 146.13 | 175.69 | 208.35 |
| Shandong | Eastern | 38.55 | 100.35 | 159.3 | 181.88 | 220.66 |
| Henan | Central | 28.4 | 83.68 | 142.08 | 166.65 | 205.34 |
| Hubei | Central | 39.82 | 101.42 | 164.76 | 190.14 | 226.75 |
| Hunan | Central | 32.68 | 93.71 | 147.71 | 167.27 | 206.38 |
| Guangdong | Eastern | 69.48 | 127.06 | 184.78 | 201.53 | 240.95 |
| Guangxi | Western | 33.89 | 89.35 | 141.46 | 166.12 | 207.23 |
| Hainan | Eastern | 45.56 | 102.94 | 158.26 | 179.62 | 230.33 |
| Chongqing | Western | 41.89 | 100.02 | 159.86 | 184.71 | 221.84 |
| Sichuan | Western | 40.16 | 100.13 | 153.04 | 173.82 | 215.48 |
| Guizhou | Western | 18.47 | 75.87 | 121.22 | 154.62 | 193.29 |
| Yunnan | Western | 24.91 | 84.43 | 137.9 | 164.05 | 203.76 |
| Xizang | Western | 16.22 | 68.53 | 115.1 | 143.91 | 186.38 |
| Shaanxi | Western | 40.96 | 98.24 | 148.37 | 178.73 | 216.12 |
| Gansu | Western | 18.84 | 76.29 | 128.39 | 159.76 | 199.78 |
| Qinghai | Western | 18.33 | 61.47 | 118.01 | 145.93 | 195.15 |
| Ningxia | Western | 31.31 | 87.13 | 136.74 | 165.26 | 214.7 |
| Xinjiang | Western | 20.34 | 82.45 | 143.4 | 163.67 | 205.49 |

**Table A2.** Regression results of explanatory variables lagged with one period.

| Variable | lnSO₂ | lnCOD | |
| --- | --- | --- | --- |
|  | FE | FE |  |
| L.Index | －0.0486**  （1.99） | －0.0376***  （3.00） |  |
| L.PGDP | 0.1713**  （2.60） | 0.1294***  （3.82） |  |
| L.$PGDP^{2}$ | －0.0169***  （4.54） | －0.0117***  （6.10） |  |
| L.Theil | 1.1396**  （2.27） | 0.6371**  （2.46） |  |
| L.Industry | 0.0934*  （1.85） | 0.0849***  （3.28） |  |
| Constant | 3.2603***  （14.09） | 3.5879***  （30.16） |  |
| R-squared | 0.6583 | 0.7669 |  |
| F-test | 2120.28*** | 5088.24*** |  |
| Hausman test | 18.45*** | 19.67*** |  |
| Obs | 155 155 | |  |

Note: t-values are reported in parentheses. * p < 0.1; ** p < 0.05; *** p < 0.01.

**Table A3.** Regression results of 2011–2014.

| Variables | lnSO₂ | lnCOD | |
| --- | --- | --- | --- |
|  | FE | FE |  |
| Index | －0.0575***  （3.22） | －0.0376***  （3.96） |  |
| PGDP | 0.0792  （1.64） | 0.0537**  （2.09） |  |
| $PGDP^{2}$ | －0.0089***  （3.27） | －0.0064***  （4.46） |  |
| Theil | 0.6151*  （1.67） | 0.1535  （0.78） |  |
| Industry | 0.0502  （0.01） | 0.0488**  （2.48） |  |
| Constant | 3.6969***  （21.82） | 3.9203***  （43.52） |  |
| R-squared | 0.6013 | 0.8812 |  |
| F-test | 4114.83 *** | 9042.96*** |  |
| Hausman test | 20.50*** | 21.03*** |  |
| Obs | 155 155 | |  |

Note: t-values are reported in parentheses. * p < 0.1; ** p < 0.05; *** p < 0.01.
